# Heat‐Treated Whey Powder Solutions as Natural Antibrowning Agents in Fresh‐Cut Potatoes

**DOI:** 10.1002/fsn3.72093

**Published:** 2026-07-07

**Authors:** Cemal Kasnak, Recep Palamutoğlu, Merve İnce‐Palamutoğlu

**Affiliations:** ^1^ Department of Nutrition and Dietetics, Faculty of Health Sciences Afyonkarahisar Health Sciences University Afyonkarahisar Türkiye

**Keywords:** antibrowning agents, enzymatic browning, phenolic compound, polyphenol oxidase, potato, whey powder

## Abstract

Enzymatic browning severely limits the quality and marketability of fresh‐cut potatoes. This study investigated the antibrowning potential of whey powder solutions (WPS) subjected to different heat treatments (20°C, 50°C, 70°C, and 90°C) and their effects on enzymatic activity, oxidative status, and nutritional quality during refrigerated storage. Among the treatments, WPS heated at 50°C (WPS 50) was the most effective in delaying browning development. WPS 50 significantly reduced polyphenol oxidase (PPO) and phenylalanine ammonia‐lyase (PAL) activities, resulting in lower accumulation of chlorogenic acid, the main PPO substrate. In addition, WPS 50 maintained higher L* values and reduced total color difference (TCD) by up to 48% compared to the control. The treatment also limited hydrogen peroxide accumulation and preserved ascorbic acid content throughout storage. Correlation analysis revealed strong associations between PPO activity, oxidative markers, phenolic metabolism, and color deterioration. The improved antibrowning performance of WPS 50 could be related to heat‐induced structural changes in whey components, particularly, changes in sulfhydryl group availability and pH characteristics. Overall, these findings highlight that moderate heat treatment (50°C) optimizes the functional properties of WPS, enabling effective suppression of enzymatic browning through coordinated regulation of enzymatic activity, oxidative status, and phenolic metabolism. Heat‐treated WPS therefore represent a promising natural alternative to conventional anti‐browning agents for fresh‐cut potato processing.

## Graphical abstract

Fresh‐cut potatoes were immersed in 5% whey powder solutions preheated to 20°C, 50°C, 70°C, or 90°C for 10 min. Moderate heat treatment (50°C) optimized whey protein functionality by increasing free sulfhydryl groups, maintaining suitable acidity, and promoting partial protein unfolding. This treatment most effectively inhibited enzymatic browning through reduced PPO and PAL activities, lowered oxidative stress (H_2_O_2_ and MDA), preserved chlorogenic acid and ascorbic acid, and maintained visual quality by increasing L* value, reducing total color difference (ΔE*), and delaying browning during refrigerated storage.

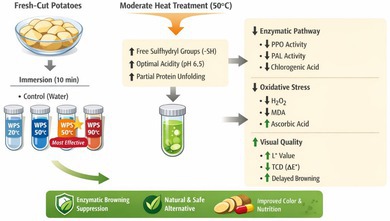

## 1 Introduction

Nutrition is defined as the provision of energy and nutrients required by an individual in adequate and balanced amounts according to age, sex, working conditions, and special circumstances (World Health Organization 2020). However, food preparation and cooking processes used to make foods consumable may reduce their beneficial properties and even cause adverse health effects when carried out under inappropriate conditions. Incorrect practices not only result in nutrient losses but also cause economic losses (McGee 2018).

Changes in lifestyle, urbanization, and increased work‐related time constraints have significantly influenced dietary habits and food consumption patterns over recent decades (Devine et al. 2009; Jabs and Devine 2006). These changes have increased consumer demand for practical, minimally processed, and ready‐to‐eat food products that require less preparation time. In this context, fresh‐cut potatoes (FCP) have emerged as an important alternative for consumers seeking convenient meal solutions (Sipahi et al. 2013). Fresh‐cut products represent a continuously expanding market segment in the food industry due to their convenience and time‐saving advantages. However, peeling and cutting operations cause tissue damage in potatoes, leading to increased respiration rates, biochemical deterioration, and enzymatic browning (Azarakhsh et al. 2014). Enzymatic browning adversely affects the color, texture, nutritional quality, and aroma of the product, thereby reducing its marketability (Saxena et al. 2012).

Phenolic compounds, which are the main substrates of enzymatic browning, play a central role in this process in FCP. In plants, phenolic compounds are primarily synthesized via the phenylpropanoid metabolic pathway. Therefore, regulation of phenylpropanoid metabolism is considered an effective strategy for delaying enzymatic browning in FCP. Studies conducted by Dai et al. (2023) demonstrated that sodium nitroprusside inhibited the activity of phenylalanine ammonia‐lyase (PAL), a key enzyme in the phenylpropanoid pathway, thereby reducing the synthesis rate of phenolic compounds and effectively controlling enzymatic browning. Similarly, treatments with exogenous proline and sodium bisulfite have been reported to reduce phenolic compound content and protect FCP against enzymatic browning by enhancing antioxidant enzyme activities (Liu et al. 2022; Ma et al. 2017). However, the application of chemical browning inhibitors remains limited due to potential food safety concerns (Tinello and Lante 2018). Therefore, the development of safe, natural, and consumer‐friendly preservation technologies capable of effectively preventing browning in FCP is of great importance from both theoretical and industrial perspectives.

Polyphenol oxidase (PPO) is a copper‐containing enzyme that oxidizes phenolic compounds to o‐quinones, initiating the enzymatic browning process. This process proceeds through phenolic oxidation, quinone formation, quinone polymerization, and the formation of brown pigments. Disruption of cellular integrity following cutting facilitates contact between PPO and phenolic substrates, thereby accelerating browning reactions. Nevertheless, the mechanism is not limited solely to PPO activity; it has been reported that the resulting quinones also contribute to browning through nonenzymatic reactions, indicating that enzymatic browning is part of a broader chemical process (Guan et al. 2024). In particular, the increased surface area of potatoes cut into small cubes accelerates the formation of quinones.

Whey, a by‐product of industrial cheese production, has significant potential in the food industry due to its high biological value and functional properties (Altunkaya and Gökmen 2009). Moreover, owing to its rich thiol (–SH) group content, whey is considered a potential inhibitor of enzymatic browning in fruits and vegetables. Numerous studies have demonstrated that sulfhydryl compounds can reduce enzymatic browning by inhibiting PPO activity (Eissa et al. 2006). However, studies investigating the effects of whey protein concentrates on enzymatic browning are limited (Altunkaya and Gökmen 2009). In addition, no studies have been found examining changes in free thiol content following exposure of whey solutions to different thermal treatments and the subsequent effects on enzymatic browning.

Accordingly, the aim of this study was to investigate the anti‐browning potential of thermally treated whey powder solutions (WPS) in FCP. In this study, the effects of FCP immersed in WPS exposed to different thermal treatments on color, enzymatic activity, and nutritional quality were evaluated during cold storage. To determine quantitative changes occurring in the product, color parameters, PPO and PAL activities, ascorbic acid (AA), malondialdehyde (MDA), hydrogen peroxide (H_2_O_2_), chlorogenic acid (CHA), caffeic acid (CA), and total phenolic content (TPC) were monitored.

## 2 Materials and Methods

### 2.1 Materials

Potatoes (*Marabel cultivar*) of the uniform size (average weight 125.42 ± 7.53 g) were taken from a local market in Afyonkarahisar province, Türkiye. The cheese serum powder was provided from Kimbiotek (Kimbiotek—Food Additives Industry and Trade Limited Company, Türkiye). The cheese serum powder was obtained by drying of cow's milk at 190°C. Potassium sodium tartrate tetrahydrate (KNaC_4_H_4_O_6_·4H_2_O), sodium carbonate (Na_2_CO_3_), sodium hydroxide (NaOH), copper(II) sulfate pentahydrate (CuSO_4_·5H_2_O), sodium hydroxide (NaOH), Folin–Ciocalteu reagent (H_3_PW_12_O_40_/H_3_PMo_12_O_40_), bovine serum albumin (BSA), 5,5′‐dithio‐bis(2‐nitrobenzoic acid) (C_14_H_8_N_2_O_8_S_2_), phenolphthalein (C_20_H_14_O_4_), sodium hydroxide (NaOH), monopotassium phosphate (KH_2_PO_4_), dipotassium hydrogen phosphate (K_2_HPO_4_), catechin hydrate (C_15_H_14_O_6_·xH_2_O), boric acid (H_3_BO_3_), l‐phenylalanine (C_9_H_11_NO_2_), trichloroacetic acid (CCl_3_COOH), thiobarbituric acid (C_4_H_4_N_2_O_2_S), potassium iodide (KI), hydrogen peroxide (H_2_O_2_), gallic acid (C_7_H_6_O_5_), methanol (CH_3_OH), sodium carbonate (Na_2_CO_3_), formic acid (HCOOH), metaphosphoric acid ((HPO_3_)_*n*_), AA (C_6_H_8_O_6_), CHA (C_16_H_18_O_9_), and CA (C_9_H_8_O_4_) were purchased from Sigma‐Aldrich.

### 2.2 Heat Treatment of WPS

In our preliminary experiment, the PPO inhibition effect of 5%, 10%, 20%, and 30% whey powder aqueous solution was investigated in FCP, and it was determined that the concentration keeping PPO the lowest was 5% WPS. The 5% WPS were held in a water bath for 15 min at 20°C, 50°C, 70°C, and 90°C. WPS were cooled down to 20°C, except for the solution kept in the water bath at 20°C. All temperatures were controlled with a food thermometer. The pH values of the 5% WPS solutions ranged between 6.12 and 6.38 depending on the heat treatment applied.

### 2.3 Sample Preparation

Potato samples brought to the laboratory were washed, dried at room temperature, peeled, and cut into cubes with a thickness of 1 cm (±0.25). Elliptical slices of 1 cm thickness from potatoes were cut to be used only for color analysis. Five different treatment groups were formed as Control (potato samples immersed in pure water), WPS 20 (solution kept at 20°C for 15 min), WPS 50 (solution kept at 50°C for 15 min and then cooled), WPS 70 (solution kept at 70°C for 15 min and then cooled), and WPS 90 (solution kept at 90°C for 15 min and then cooled), respectively. The FCP were immersed in these solutions for 10 min, as preliminary trials indicated that this immersion time provided the most effective preservation of sample quality and browning inhibition. Then, fresh cubic potatoes dried under room conditions for 10 min were placed on polyethylene plates and covered with stretch film. Finally, the prepared samples were stored at 4°C and 95% relative humidity for Day 9 in a refrigerator and used for experimental measurements on Days 3, 6, and 9. Each treatment consisted of two replicates, and 10 pieces obtained from different potato tubers were used for each experiment.

### 2.4 Determination of Protein, Free Sulfhydryl (–SH) Groups and Titratable Acidity (TA) of WPS

The Hartree–Lowry method was used to determine the total water‐soluble protein (Olson 2016). One milliliter of sample and 0.9 mL of Hartree–Lowry Reagent A (potassium sodium tartrate tetrahydrate: sodium carbonate: sodium hydroxide 1 N, 0.2:10:50, w/w/v in water) were mixed and left in the water bath (Nuve BM 402, Türkiye) to incubate at 50°C for 10 min. Then, 0.1 mL Hartree–Lowry Reagent B (potassium sodium tartrate tetrahydrate: Copper (II) sulfate pentahydrate: Sodium hydroxide 1 N, 2:1:100, w/w/v in water) was added and incubated at room temperature for 10 min. Finally, 3 mL of Hartree–Lowry Reagent C (Folin‐Ciocalteau reagent: water, 1 mL:15 mL) was added and the mixture was incubated for a further 10 min in a 50°C water bath and read at 650 nm on the spectrophotometer (Optizen pop, Korea) against the blank. Results were determined as g BSA/kg fresh weight with the help of the BSA standard curve (*R* = 0.9940).

Free sulfhydryl (–SH) groups were determined according to (Melo and Hansen 1978). Ten milliliters of sample and 30 mL of 10 mM 5,5‐dithio‐bis (2‐nitrobenzoic acid) (dissolved in 0.1 M phosphate buffer (pH 7.0)) were mixed. The mixture was left for 30 min and centrifuged at 3007*g* for 30 min. The absorbance of the supernatant was read with a spectrophotometer at 412 nm. The extinction coefficient was calculated using (*e* = 13.600 M/L^/^cm), and the results were expressed in μmol/L.

TA was determined according to Liu et al. (2020). Whey powder (0.5 g) was added into 10 mL of distilled water and mixed. Then 2–3 drops of the indicator phenolphthalein were added. The color was titrated with 0.1 N NaOH until it turned slightly pink. The results were expressed in g lactic acid/L.

### 2.5 Determination of PPO and PAL Activities of Potatoes

PPO extraction was performed according to the method described by Kasnak (2020). Potato samples were finely chopped using a knife, and 2 g of the chopped sample was accurately weighed. Cold phosphate buffer (10 mL, pH 6.5, 4°C) was added to the samples, which were then homogenized in an ice–water bath at 11,000 rpm for 3 min. Following homogenization, the samples were centrifuged at 12,000 rpm for 25 min at 4°C. The supernatant was collected and filtered to obtain the crude enzyme extract. For PPO activity determination, 0.25 mL of the crude extract was mixed with 0.75 mL of phosphate buffer (0.1 M, pH 5.8) and 0.50 mL of catechin hydrate solution (20 mM), and the mixture was incubated at room temperature for 30 min. A buffer solution was used as the blank instead of the crude enzyme extract. Absorbance was measured at 410 nm using a UV/Vis spectrophotometer (Optizen POP, Korea). One unit of PPO activity was defined as the amount of enzyme required to increase the absorbance by 0.001 per minute.

PAL activity was determined according to Gao, Zeng, et al. (2018) and Gao, Wu, et al. (2018), with slight modifications. By taking 1 g of the samples, it was homogenized in 9 mL of 0.1 M borate buffer (pH 8.85) for 3 min at 11,000 rpm and centrifuged at 3007*g* for 25 min at 4°C. Then the supernatant was filtered; 0.2 mL supernatant, 1.28 mL borate buffer, and 0.4 mL 50 mM phenylalanine were mixed and read at 290 nm in quartz cuvettes in the spectrophotometer. After 30 min, the mixture was read again and the difference between them was determined. The results were expressed with the unit of (U/g) fresh weight as the amount of enzyme needed to increase the absorbance by 0.001 per minute by creating new products.

### 2.6 Determination of MDA and H_2_O_2_ Contents of Potatoes

MDA was determined according to Zheng et al. (2019) with slight modifications. Taking 1 g of the samples, 10 mL of 1 g/L of precooled trichloroacetic acid was homogenized at 11,000 rpm for 3 min, centrifuged at 3007*g* for 10 min at 4°C and the supernatant was filtered. 1.2 mL filtrate and 2 mL 6.7 g/L thiobarbituric acid were put into glass tubes and mixed with vortex, then incubated at 95°C for 20 min. The mixture was quickly cooled and read in a spectrophotometer using multiple wavelengths (450, 532, 600 nm). The formula was used to calculate MDA content:

(1) $$\mathrm{MDA}\;\text{content}\;\left(\text{μmol}/\mathrm{kg}\right)=6.45\times \left({\mathrm{OD}}_{532}–{\mathrm{OD}}_{600}\right)-0.56\times {\mathrm{OD}}_{450}$$

The H_2_O_2_ content was determined according to Zheng et al. (2019), with slight modifications. One gram sample was homogenized in 9 mL of 1 g/L pre‐cooled trichloroacetic acid for 3 min at 11,000 rpm, centrifuged at 3007*g* for 10 min at 4°C and then filtered. 0.6 mL of filtrate, 1 mL of 1 M potassium iodide and 1 mL of 10 mM potassium phosphate buffer (pH 7) were mixed and read at 390 nm on the spectrophotometer against the blank. Results were determined as g H_2_O_2_/kg fresh weight with the help of the H_2_O_2_ standard curve (*R* = 0.99).

### 2.7 Determination of the Total Phenolic and Phenolic Acid Content of Potatoes

Extraction was done according to Palamutoğlu (2020). Below analyses based on extraction were made on a fresh weight basis. TPC was determined according to Kaur and Kapoor (2002). A mixture consisting of 0.25 mL of the extraction solution, 0.25 mL of Folin–Ciocalteu reagent, and 3.5 mL of deionized water was prepared and incubated for 3 min. Then 1 mL of 20% Na_2_CO_3_ was added and mixed again. The mixture was left in the water bath at 25°C for 1 h. Absorbance was measured at 765 nm using a UV–visible spectrophotometer (Optizen pop, Korea). The results are given in mg gallic acid/kg fresh weight (*R* = 0.9954).

Phenolic compounds were determined according to the method described by Albishi et al. (2013) with slight modifications. High‐performance liquid chromatography (HPLC) analysis was carried out using a Thermo Scientific Dionex Ultimate 3000 HPLC system equipped with an autosampler (WPS‐3000SL), column compartment (TCC‐3000SD), and multiple wavelength photodiode array detector (MWD‐3000). Separation was achieved using an SB‐C18 column (250 × 4.6 mm, 5 μm particle size). The mobile phase consisted of 1% formic acid in purified water (solvent A) and 100% methanol (solvent B). The gradient elution program was applied as follows: 5% B (0–5 min), 50% B (5.1–10 min), 70% B (10.1–15 min), 80% B (15.1–20 min), and 100% B (20.1–25 min). Before injection, methanolic extracts were filtered through a 0.45 μm nylon membrane filter, and 20 μL of the filtrate was injected into the system. The flow rate was maintained at 1.5 mL/min, the column temperature was set at 35°C, and chromatograms were monitored at 320 nm. Calibration curves were constructed using CHA and CA standards at concentrations of 30, 40, 48, 60, 80, 100, and 120 μg/mL. The calibration curves exhibited excellent linearity, with coefficients of determination (*R* ^2^) of 0.9984 and 0.9990 for CHA and CA, respectively.

### 2.8 Determination of AA Content and Color of Potatoes

Two gram sample was homogenized for 3 min at 11,000 rpm in 10 mL of 2.5% metaphosphoric acid solution (metaphosphoric acid: glacial acetic acid: water, 2.5, 1, 100 w/v/v). Then it was centrifuged at 3007*g* for 10 min, and the collected supernatant was filtered. The filtrate (20 μL) was injected into HPLC without waiting. Thermo Scientific HPLC Dionex Ultimate 3000 was equipped with a PDA detector. The separation was carried out with isocratic elution with 2% KH_2_PO_4_ (w/v) at pH 2.5 (adjusted with metaphosphoric acid solution) for 30 min. A C18 column (250 × 4.6 mm ID, 5 μm particle size) was used. The flow rate was 0.5 mL/min at 30°C, and peaks were read at 244 nm. The calibration curve for AA was prepared using standard solutions at concentrations of 40.0, 53.3, 64.0, 80.0, 107.0, and 160.0 μg/mL. The calibration curve exhibited excellent linearity with a coefficient of determination (*R* ^2^) of 0.9969.

Elliptical potato slices were used for color measurements. Color parameters were determined at 0, 3, 6, and 9 days of storage using a Ci64 color spectrophotometer (X‐Rite, USA). The instrument was calibrated prior to analysis. Color values were expressed according to the Hunter color system as L* (lightness; 0 = black, 100 = white), a* (green [−] to red [+]), and b* (blue [−] to yellow [+]). Changes in sample color during storage were evaluated based on variations in L*, a*, and b* values. In addition, the total color difference (TCD, ΔE*) was calculated using the following equation:

(2) $${\mathrm{\Delta E}}^{*}=\sqrt{{\Delta L}^{*2}+{\Delta a}^{*2}+{\Delta b}^{*2}}$$

### 2.9 Statistical Analysis

All analyses were performed using SPSS Statistics version 26.0 (IBM Corp., Armonk, NY, USA). Results were presented as mean ± standard deviation. Differences among treatments were evaluated by one‐way analysis of variance (ANOVA), followed by Duncan's multiple range test for post hoc comparisons at a significance level of *p* < 0.05. Pearson's correlation analysis was additionally conducted to assess the relationships among the dependent variables.

## 3 Results and Discussion

### 3.1 Chemical Properties of Thermally Treated WPSs

Table 1 shows the protein, thiol, and titration acidity content of whey solutions kept in a water‐bath at different temperatures. There is no significant difference between protein amounts of WPS samples (*p* > 0.05). However, heating of the WPS samples caused significant changes in the amount of thiol compounds between the groups (*p* < 0.01). The highest thiol content was observed in WPS 70, whereas the lowest thiol content was observed in WPS 90. This revealed that heating up to 70°C increased thiol compounds, and high temperatures such as 90°C decreased thiol compounds. Thiol compounds are effective in preventing browning reactions caused by PPO in fruit and vegetables (Eissa et al. 2006). Significant changes were observed in the TA as a result of heating the WPS samples (*p* < 0.01). The highest TA was observed in WPS 20; TA decreased as WPS was heated.

**TABLE 1:** Physicochemical properties of whey powder aqueous solutions kept in water bath at different temperatures.

Treatments	Protein (g/L)	Sulfhydryl groups (μmol/L)	Titratable acidity (g/L)
WPS 20	9.30 ± 0.07^a^	20.33 ± 0.01^c^	6.80 ± 0.06^a^
WPS 50	8.60 ± 0.04^a^	22.17 ± 0.02^b^	4.50 ± 0.06^b^
WPS 70	8.80 ± 0.09^a^	25.55 ± 0.02^a^	2.30 ± 0.03^c^
WPS 90	9.30 ± 0.11^a^	18.60 ± 0.07^d^	2.30 ± 0.01^c^

*Note:* Data represent the mean (*n* = 2) ± SD. Different letters show the significant differences (*α* = 0.05) on WPS treatments of same physicochemical property according to the Duncan multiple comparison test (*p* < 0.05).

Abbreviations: WPS 20: whey powder solution kept at 20°C, WPS 50: whey powder solution kept at 50°C, WPS 70: whey powder solution kept at 70°C, WPS 90: whey powder solution kept at 90°C.

### 3.2 Effect of WPS on PPO and PAL Activities of Potatoes

The occurrence of browning results from the enzymatic oxidation of phenolic compounds to o‐quinones. This reaction is carried out by the PPO enzyme, which is very reactive and forms brown polymers (Franck et al. 2007). Table 2 shows the significant correlations of all analyzed values with each other. PPO activity showed a strong positive correlation with H_2_O_2_, CHA, TPC and a*, TCD values. In contrast, PPO activity was negatively correlated with b* values, suggesting that increased enzymatic browning was associated with reduced yellowness and overall color quality deterioration in FCP (Table 2).

**TABLE 2:** Significant correlations of all analyzed values (*n* = 40).

Correlations		PPO	PAL	H_2_O_2_	MDA	TPC	CHA	CA	AA	L*	a*	b*	TCD
PPO	Pearson's correlation	1	0.246	0.697**	−0.535**	0.610**	0.622**	0.197	−0.185	−0.751**	0.747**	−0.660**	0.742**
	Sig.		0.126	0.000	0.000	0.000	0.000	0.223	0.253	0.000	0.000	0.000	0.000
PAL	Pearson's correlation	0.246	1	0.099	−0.209	0.313*	0.654**	0.325*	−0.146	−0.294	0.594	−0.208	0.249
	Sig.	0.126		0.541	0.195	0.049	0.000	0.041	0.370	0.066	0.000	0.198	0.122
H_2_O_2_	Pearson's correlation	0.697**	0.099	1	−0.196	0.318*	0.474**	−0.078	−0.173	−0.570**	0.447**	−0.457**	0.540**
	Sig.	0.000	0.541		0.225	0.046	0.002	0.631	0.285	0.000	0.004	0.003	0.000
MDA	Pearson's correlation	−0.535**	−0.209	−0.196	1	−0.526**	−0.276	−0.525**	−0.126	0.410**	−0.530**	0.461**	−0.465**
	Sig.	0.000	0.195	0.225		0.000	0.085	0.001	0.438	0.009	0.000	0.003	0.003
TPC	Pearson's correlation	0.610**	0.313*	0.318*	−0.526**	1	0.655**	0.528**	0.126	−0.462**	0.566**	−0.389**	0.428**
	Sig.	0.000	0.049	0.046	0.000		0.000	0.000	0.440	0.003	0.000	0.013	0.006
CHA	Pearson's correlation	0.622**	0.654**	0.474**	−0.276	0.655**	1	0.358*	−0.129	−0.569**	0.741**	−0.473**	0.543**
	Sig.	0.000	0.000	0.002	0.085	0.000		0.023	0.429	0.000	0.000	0.002	0.000
CA	Pearson's correlation	0.197	0.325*	−0.078	−0.525**	0.528**	0.358*	1	0.105	−0.115	0.505**	−0.222	0.205
	Sig.	0.223	0.041	0.631	0.001	0.000	0.023		0.520	0.481	0.001	0.168	0.205
AA	Pearson Correlation	−0.185	−0.146	−0.173	−0.126	0.126	−0.129	0.105	1	0.567**	−0.340*	0.269	−0.331*
	Sig.	0.253	0.370	0.285	0.438	0.440	0.429	0.520		0.000	0.032	0.093	0.037
L*	Pearson's correlation	−0.751**	−0.294	−0.570**	0.410**	−0.462**	−0.569**	−0.115	0.567**	1	−0.783**	0.664**	−0.790**
	Sig.	0.000	0.066	0.000	0.009	0.003	0.000	0.481	0.000		0.000	0.000	0.000
a*	Pearson's correlation	0.747**	0.594	0.447**	−0.530**	0.566**	0.741**	0.505**	−0.340*	−0.783**	1	−0.671**	−0.790**
	Sig.	0.000	0.000	0.004	0.000	0.000	0.000	0.001	0.032	0.000		0.000	0.000
b*	Pearson's correlation	−0.660**	−0.208	−0.457**	0.461**	−0.389**	−0.473**	−0.222	0.269	0.664**	−0.671**	1	−0.790**
	Sig.	0.000	0.198	0.003	0.003	0.013	0.002	0.168	0.093	0.000	0.000		0.000
TCD	Pearson's correlation	0.742**	0.249	0.540**	−0.465**	0.428**	0.543**	0.205	−0.331*	−0.790**	0.756**	−0.969**	1
	Sig.	0.000	0.122	0.000	0.003	0.006	0.000	0.205	0.037	0.000	0.000	0.000	

Abbreviations: AA: ascorbic acid, CA: caffeic acid, CHA: chlorogenic acid, H_2_O_2_: hydrogen peroxide, MDA: malondialdehyde, PAL: phenyl ammonia lyase, PPO: polyphenol oxidase, TCD: total color differences, TPC: total phenolic content.

* Correlation is significant at the 0.05 level.

** Correlation is significant at the 0.01 level.

**FIGURE 1:**
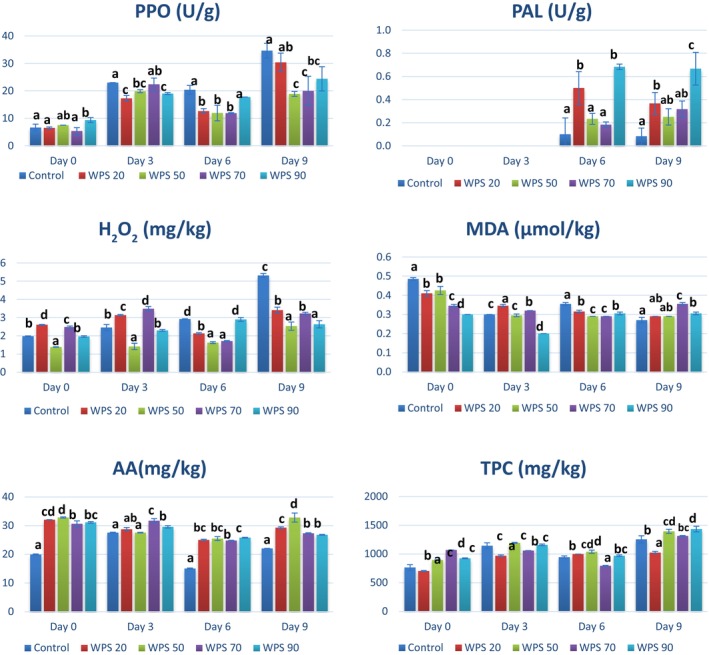
The effect of WPS heated at different temperatures on PPO, PAL, H_2_O_2_, MDA, AA, and TPC of FCP. Different lowercase letters indicate significant differences (*α* = 0.05) according to the Duncan multiple comparison test (*p* < 0.05). Error bars represent standard deviations of the mean. AA: ascorbic acid, H_2_O_2_: hydrogen peroxide, MDA: malondialdehyde, PAL: phenyl ammonia lyase, PPO: polyphenol oxidase, TPC: soluble phenolic content, WPS 20: whey powder solution kept at 20°C, WPS 50: whey powder solution kept at 50°C, WPS 70: whey powder solution kept at 70°C, WPS 90: whey powder solution kept at 90°C.

PPO activity showed a strong negative correlation with MDA, L*, and b* values. Figure 1 presents the changes in PPO and PAL activities of whey protein solution treatments exposed to different temperatures. At the end of storage, WPS 20, WPS 50, WPS 70, and WPS 90 treatments significantly reduced PPO activity compared to the control group (*p* < 0.05). Among the treatments, WPS 50 showed the lowest PPO activity. On Day 9, PPO activity in the WPS 50 group was 54.5% of that observed in the control group, followed by WPS 70, which showed 57.8% of the control PPO activity. These findings may be associated with heat‐induced structural modifications in whey proteins at 50°C and 70°C, which could increase the availability of bioactive peptides and sulfhydryl‐containing compounds. Thermal treatment is known to induce conformational changes in major whey proteins, particularly β‐lactoglobulin, leading to partial unfolding and increased exposure of buried sulfhydryl groups and hydrophobic regions. Moderate heating may also enhance the release or accessibility of bioactive peptide regions, whereas excessive heating can promote irreversible aggregation and reduce the functional reactivity of whey components. Peptides may act as PPO inhibitors by interacting with phenolic substrates and stabilizing o‐quinones, thereby limiting melanin formation and surface color changes (da Silva et al. 2018). Collagen peptide and AA binary treatment have been shown to slow PPO activity (Kasnak 2020). Besides, the increase in free thiol content observed in WPS exposed to 50°C and 70°C may be associated with reduced enzymatic browning. Compounds containing thiol react with o‐quinone to produce stable, colorless adducts instead of brown pigments, thereby limiting enzymatic browning (Eissa et al. 2006). However, although WPS 70 exhibited the highest sulfhydryl content, PPO activity did not differ significantly between WPS 50 and WPS 70 treatments throughout storage. Nevertheless, WPS 50 showed superior color preservation compared with WPS 70. These findings suggest that browning control cannot be explained solely by PPO inhibition, but rather by the combined influence of several factors involved in browning reactions, including enzymatic activity, substrate availability, sulfhydryl group content, phenolic composition, and pH‐related properties. The highest TA was observed in WPS 20, whereas acidity gradually decreased with increasing heat treatment temperature. Moderate heating at 50°C may therefore have provided a more favorable balance between sulfhydryl availability and acidity, potentially improving the interaction of reactive whey components with PPO and o‐quinones. In contrast, excessive heating at 70°C may have promoted protein aggregation and reduced the functional accessibility of reactive sulfhydryl groups despite their higher measured concentration. Similar observations have been reported in studies showing that the antibrowning activity of protein‐based systems depends not only on thiol content but also on protein conformation and environmental conditions affecting enzyme interactions (da Silva et al. 2018; Friedman and Bautista 1995).

PAL is an enzyme that catalyzes the conversion of l‐phenylalanine into ammonia and transcinnamic acid. It plays an important role in the biosynthesis of polyphenolic compounds such as flavonoids, phenylpropanoids, and lignin, particularly, as part of the defense response in damaged plant tissues (Tanaka et al. 1989). Consistent with this mechanism, PAL activity showed positive correlations with CHA, CA, and TPC (Table 2).

As seen in Figure 1, PAL activity was not observed unexpectedly in all samples in the first Day 3. PAL activity of WPS 20 and WPS 90 increased significantly on Day 6 compared to control (*p* < 0.05). On Day 9, while PAL activity of WPS 20, WPS 90, and control showed a slight decrease, PAL activity of WPS 50 and WPS 70 increased. At the end of the storage, WPS 50 and WPS 70 not only kept PPO activity low but also kept PAL activity significantly lower (*p* < 0.05). Similarly, the inhibition of enzymatic browning in FCP through modulation of PPO and related enzymatic activities has been demonstrated using different food additives such as AA and l‐cysteine, highlighting that enzymatic browning can be controlled by targeting key enzymes involved in phenolic metabolism (Li et al. 2023). In this context, the present study demonstrates that heat‐treated WPS represent an alternative strategy capable of regulating PPO and PAL activities to mitigate enzymatic browning. Consistent with previous findings, another study showed that PAL activity was very low on the first day in fresh cut Chinese water chestnut slices and reached its highest values on Day 6 (Peng et al. 2008). PAL activity decreased over the next 12 days. Melatonin treatment on freshly cut pears has been shown to increase PAL activity compared to control throughout the entire storage (Zheng et al. 2019).

### 3.3 Effect of WPS on H_2_O_2_ and MDA Contents of Potatoes

H_2_O_2_ accelerates the breakdown of the cell membrane and causes browning in the pericarp of chopped fruit and vegetables (Lin et al. 2017). In parallel with this view, H_2_O_2_ showed a strong negative correlation with L* value and positive correlations with CHA, TPC, and TCD values (Table 2). The H_2_O_2_ content in the control group gradually increased during storage and reached the highest level (5.32 g/kg) among all samples at the end of storage (Figure 1). Among the treatments, WPS 50 maintained comparatively lower H_2_O_2_ levels throughout storage, with the lowest final value (2.53 g/kg) observed on Day 9. Compared with the control, the WPS 50 group showed significantly lower H_2_O_2_ contents from Days 0 to 9 (*p* < 0.05), corresponding to reductions of 30%, 42%, 44%, and 52% on Days 0, 3, 6, and 9, respectively. Although fluctuations in H_2_O_2_ content were observed in the other WPS treatments, all treatment groups exhibited significantly lower H_2_O_2_ levels than the control at the end of storage (*p* < 0.05). Similarly, yogurt serum treatment has been reported to reduce PPO activity and reactive oxygen species such as H_2_O_2_ in FCP, highlighting the role of protein/serum‐derived matrices in modulating enzymatic and oxidative browning pathways (Kasnak and Palamutoglu 2021). Li et al. (2019) reported that cold plasma processing in fresh‐cut pitaya fruit significantly increased H_2_O_2_ production compared to the control group. Gao, Zeng, et al. (2018) and Gao, Wu, et al. (2018) reported that a significant decrease in H_2_O_2_ concentration in fresh‐cut apples treated with gamma aminobutyric acid compared to negative control after 5‐day storage (*p* < 0.05).

Lipid peroxidation is considered a major indicator of membrane system damage and impairment of cellular metabolism. Since MDA is a product of lipid peroxidation in the cell membrane, it can directly reflect the degree of membrane lipid peroxidation (Ma et al. 2016). MDA showed negative correlations with CA, TPC, and TCD, whereas a positive correlation was observed with the L* value (Table 2). During storage, the lowest average MDA content was observed in the WPS 90 group, followed by WPS 50, WPS 70, WPS 20, and the control group. The MDA contents of potatoes subjected to WPS treatments are presented in Figure 1. A fluctuating trend was observed in the MDA content of the control group throughout storage. Initially, the MDA content of the control group was significantly higher than those of the WPS‐treated groups (*p* < 0.05). This finding may be associated with the absence of a protective treatment in the control group, which could have increased susceptibility to membrane disruption and lipid peroxidation.

The MDA content of the WPS 90 treatment was significantly lower compared to the control and other treatments the first Day 3 (*p* < 0.05). Although the MDA content of the WPS 50 treatment was initially higher than other WPS treatments, it kept the MDA content both low and stable during the storage period. Gao, Zeng, et al. (2018) and Gao, Wu, et al. (2018) reported that the MDA content of cut potatoes treated with gamma aminobutyric acid was found lower on Days 4 and 5 and higher at the end of storage compared to the control. Contrary to our results, Liu et al. (2019) reported that the MDA content of FCP treated with purslane extract increased over the entire 8‐day storage. Xylia et al. (2019) reported that the MDA content of the samples treated with *Origanum majorana* essential oil, marjoram hydrosol, and AA in grated carrot was higher than the control after Day 9 of storage.

### 3.4 Effect of WPS on TPC and Phenolic Acid Contents of Potatoes

Subjecting the potatoes to mechanical processes such as peel, cut, and slicing causes the parenchyma cells to break down and release the PPO. This enzyme reduces the phenolic compounds such as CHA and CA and forms o‐quinones that cause browning color formation (Kasnak 2020). CHA, the major phenolic compound in potatoes, is considered an important substrate involved in PPO‐catalyzed enzymatic browning (Cantos et al. 2002). In the present study, CHA showed strong positive correlations with PPO and PAL activities (Table 2). In addition, CHA was positively correlated with CA, TPC, and TCD, whereas a negative correlation was observed with the L* value. Similar associations between phenolic compounds and browning‐related parameters have also been reported in studies investigating phenolic‐rich natural extracts in FCP. In a recent study, *Quercus ithaburensis* cupule extract was reported to effectively reduce enzymatic browning by regulating PPO activity and phenolic metabolism, highlighting the role of phenolic compounds in color stability (Palamutoğlu and Kasnak 2024). In line with these findings, the present study showed that heat‐treated WPS modulated phenolic acid accumulation, particularly CHA, and contributed to the control of enzymatic browning through combined effects on enzyme activity and phenolic balance. WPS 50 has the lowest CHA level according to the average of the measurements made during storage and differs statistically significantly compared to the control (*p* < 0.05). It was followed by WPS 20, Control, WPS 90, and WPS 70 respectively. The effects of the time of storage on the levels of CHA and CA of tubers of control and treated samples are given in Table 3. Compared to the level at the start of storage, the levels of CHA increased with increasing storage duration (except WPS 70 on 3‐day) in the control and treated samples (Table 3). The concentrations of CHA for the control sample and treated samples showed significant differences on Day 3 (*p* < 0.05). The differences became more pronounced for all samples Day 9. The CHA concentrations between the samples differed significantly on the start, 3rd, 6th, and 9th days.

**TABLE 3:** Effect of heated WPS on the phenolic acid content of cut potatoes during storage.

Control and treatments	Phenolic acids (mg/kg)	Storage time			
		Day 0	Day 3	Day 6	Day 9
Control	Chlorogenic acid	58.30 ± 1.70^dC^	130.40 ± 1.98^dA^	145.90 ± 2.69^bB^	222.50 ± 3.82^dE^
	Caffeic acid	0.10 ± 0.00^dD^	0.70 ± 0.06^dE^	2.80 ± 0.23^aD^	1.90 ± 0.16^dD^
WPS 20	Chlorogenic acid	48.60 ± 2.69^dD^	111.40 ± 2.26^dB^	114.90 ± 3.25^cC^	260.20 ± 4.67^cD^
	Caffeic acid	0.80 ± 1.70^dB^	1.50 ± 1.70^dE^	3.10 ± 1.70^bD^	1.30 ± 1.70^dE^
WPS 50	Chlorogenic acid	78.30 ± 2.55^dB^	99.90 ± 2.83^dC^	102.30 ± 1.84^cD^	216.20 ± 4.10^dE^
	Caffeic acid	0.50 ± 0.07^dC^	4.80 ± 0.4^dC^	6.20 ± 0.54^bB^	11.00 ± 0.72^aA^
WPS 70	Chlorogenic acid	97.50 ± 1.84^dA^	74.20 ± 1.70^dE^	146.50 ± 2.83^bB^	342.90 ± 4.53^aA^
	Caffeic acid	0.10 ± 0.01^dD^	3.40 ± 0.24^dD^	4.60 ± 0.38^bC^	6.20 ± 0.57^bB^
WPS 90	Chlorogenic acid	74.70 ± 1.27^dB^	91.30 ± 1.98^dD^	203.60 ± 3.11^bA^	289.70 ± 4.95^bB^
	Caffeic acid	1.00 ± 0.17^dB^	7.50 ± 0.61^bB^	5.10 ± 0.58^bBC^	3.50 ± 0.37^cC^

*Note:* Data represent the mean (*n* = 2) ± SD. Different lower‐case letters show the significant differences (*α* = 0.05) on storage time of same control or treated sample according to the Duncan multiple comparison test (*p* < 0.05). Different capital letters show the significant differences (*α* = 0.05) on treatments of same storage time according to the Duncan multiple comparison test (*p* < 0.05).

Abbreviations: WPS 20: whey powder solution kept at 20°C, WPS 50: whey powder solution kept at 50°C, WPS 70: whey powder solution kept at 70°C; WPS 90, whey powder solution kept at 90°C.

Significant differences were observed in CA concentrations of all samples on Day 6 of storage compared to the beginning of storage (*p* < 0.05). CA concentrations of WPS 50 and WPS 70 continued to increase on Day 9, showing deep differences according to start level. CA concentrations of control, WPS20 and WPS90 decreased on Day 9 compared to Day 6. The study on fresh‐cut apples by Chen et al. (2016) reported that the CHA and CA content of the control sample was higher than citric acid, UV and citric acid‐UV treatments. But after Day 15 of storage, it showed that CHA and CA content decreased in control and increased in citric acid, UV, and citric acid‐UV treatments. Wang et al. (2015) reported that in the fresh‐cut slices of curing treated potatoes, CHA amounts increased dramatically from Days 0 to 12, almost doubling on Day 12 compared to the Day 9 sample.

In the TPC method, the yellow‐colored Folin–Ciocalteu reagent, which is an oxidant, takes an electron from antioxidant and turns blue. The degree of color change is proportional to the antioxidant concentration. TPC was negatively correlated with the L* value, whereas a positive correlation was observed with TCD (Table 2). The effect of treatment and storage time on the TPC is shown in Figure 1. The amount of TPC at the end of Day 9 of storage of all samples increased compared to the start level. The TPC values of the samples showed a significant difference in Day 3 compared to the fresh samples, and the difference became evident at the end of the storage (*p* < 0.05). At the end of the storage, the highest TPC value was seen on WPS 90, followed by WPS 50. The lowest TPC value was observed in WPS20. Although WPS 50 exhibited comparatively lower phenolic acid levels, its TPC values remained relatively high during storage. This finding may be associated with the higher AA content observed in the WPS 50 treatment, since the Folin–Ciocalteu reagent is not specific only to phenolic compounds and can also react with reducing substances such as vitamin C (Prior et al. 2005). Therefore, the elevated TPC values observed in WPS 50 may partially reflect the contribution of AA to the overall reducing capacity measured by the assay. Similarly (Liu et al. 2019) reported that the phenolic compound content tended to increase in fresh‐cut potato slices treated with purslane extract during storage.

### 3.5 Effect of WPS on AA and Color of Potatoes

AA is effective in preventing melanin formation due to its ability to reduce o‐quinones to o‐diphenol precursors (Cantos et al. 2002). The content of AA in FCP in response to different WPS treatments is given in Figure 1. At the beginning of the storage, the amount of AA in control remained quite low compared to WPS treatments. According to the control, the higher amount of AA in WPS treatments may be due to the AA content in cheese whey powder. Highly significant differences emerged between groups at the end of the storage (*p* < 0.001). WPS 50 had the highest AA content (32.82 mg/kg) at the end of storage, while the control had the lowest AA content (22.07 mg/kg). Saleh et al. (2013) reported that the fresh‐cut okra treated with vitamin C had L* value of 23.85% higher than the control at the end of 8 days of storage. In our study, we found a positive correlation between AA—L* value and found a negative correlation between AA—a* value (Table 2). However, contrary to expectations, the correlation coefficients between AA content and other parameters of browning were very weak and not statistically significant. This result is consistent with the previous study reporting the same lack of correlation in potato tubers during storage (Cantos et al. 2002).

**FIGURE 2:**
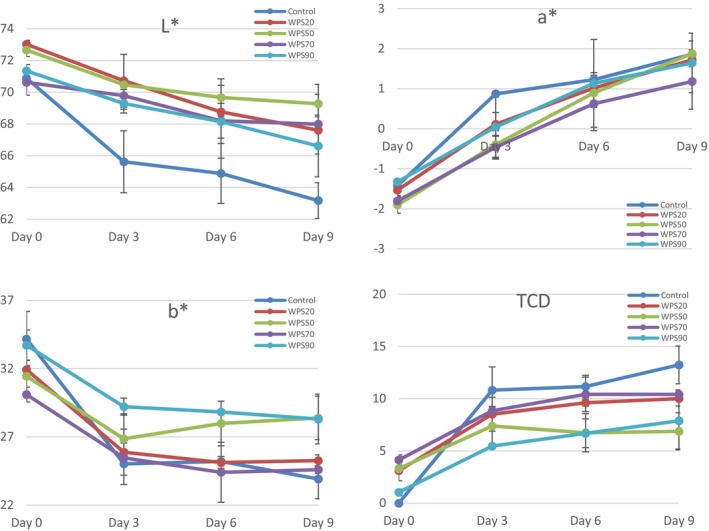
The effect of WPS heated at different temperatures on color of FCP. Error bars represent standard deviations of the mean. TCD: total color differences, WPS 20: whey powder solution kept at 20°C, WPS 50: whey powder solution kept at 50°C, WPS 70: whey powder solution kept at 70°C, WPS 90: whey powder solution kept at 90°C.

Figure 2 shows L*, a*, b* and TCD of the FCP. While the L* values of all samples decreased significantly during storage, values of TCD showed a significant increase. At the end of the storage, the L* and b* values of WPS 50 and WPS 90 were significantly higher than the control (*p* < 0.05) and the TCD values of WPS 50 and WPS 90 were significantly lower than the control (*p* < 0.05). After Day 9 of storage, there was no significant change between groups in terms of a* value (*p* > 0.05). The decrease in L* value and the increase in TCD value are indicators of enzymatic browning. While PPO activity showed strong negative correlations with L* and b* values, strong positive correlations were observed with a* and TCD values. During storage, WPS 50 and WPS 90 treatments were associated with a slower decline in L* values and a lower increase in TCD values compared with the control group. The strong negative correlation between L* and a* values further indicates that the development of darker and redder surface coloration occurred simultaneously during storage. Moreover, the very strong negative correlation between b* and TCD values suggests that loss of yellowness was closely associated with the overall visual browning development of FCP. Similarly, quercetin has been reported to effectively inhibit enzymatic browning in FCP, demonstrating that natural antioxidants can enhance color stability by regulating PPO activity and phenolic compound metabolism (Kasnak 2022). In this context, the present study shows that heat‐treated WPS not only provide antioxidant protection but also modulate enzymatic activity and phenolic balance, thereby effectively controlling enzymatic browning in FCP. Chen et al. (2016) reported that the L* value of fresh‐cut apples treated with UV and citric acid decreased significantly during storage and reported a significant increase in a* value of all apple samples stored at 5°C for Day 15.

Taken together, the present findings suggest that the anti‐browning effect of WPS 50 was not associated with a single mechanism, but rather with the combined modulation of enzymatic activity, oxidative status, phenolic metabolism, and color stability. The coordinated reductions in PPO activity, H_2_O_2_ accumulation, CHA content, and TCD values, together with the preservation of L* and AA levels, indicate that moderate thermal treatment of whey powder may create a physicochemical environment less favorable for enzymatic browning development in FCP.

## 4 Conclusion

This study demonstrates that moderate heat treatment of WPS at 50°C significantly enhances their anti‐browning functionality, enabling coordinated regulation of enzymatic activity, oxidative stress, and phenolic metabolism in FCP. Among the tested treatments, WPS 50 was the most effective in suppressing enzymatic browning by maintaining low PPO and PAL activities, limiting hydrogen peroxide accumulation, and reducing the availability of CHA, the main PPO substrate.

The superior performance of WPS 50 may be associated with an optimal balance between heat‐induced thiol availability and TA, which may contribute to improved oxidative stability and reduced enzymatic browning. Although higher thiol contents were observed at 70°C, the lower acidity at this temperature may have reduced overall antibrowning efficiency, highlighting the importance of combined physicochemical effects rather than single factors.

Overall, heat‐treated whey powder represents a safe, natural, and cost‐effective alternative to conventional antibrowning agents. Given its strong potential for application in the fresh‐cut fruit and vegetable industry, future studies should focus on elucidating the structure–function relationships of heat‐modified whey components and extending this strategy to other minimally processed commodities.

Further studies involving direct PPO inhibition assays and enzyme kinetics analyses are needed to better elucidate the mechanisms underlying the anti‐browning activity of heat‐treated WPS.

## Author Contributions

**Cemal Kasnak:** conceptualization, methodology, formal analysis, writing – review and editing, writing – original draft, investigation, supervision, validation. **Recep Palamutoğlu:** conceptualization, methodology, investigation, formal analysis, supervision, validation, writing – review and editing, writing – original draft. **Merve İnce‐Palamutoğlu:** writing – original draft, writing – review and editing.

## Funding

The authors have nothing to report.

## Disclosure

Author Approval and Responsibility Statement: All authors have read and approved the final version of the manuscript. Cemal KASNAK had full access to all of the data in this study and takes complete responsibility for the integrity of the data and the accuracy of the data analysis.

## Ethics Statement

The authors have nothing to report.

## Conflicts of Interest

The authors declare no conflicts of interest.

## Data Availability

The data that support the findings of this study are available from the corresponding author upon reasonable request.
